# Students’ experiences of using a writing-intense programme to facilitate critical thinking skills on an online clinical training platform: A pilot study

**DOI:** 10.4102/sajcd.v69i2.919

**Published:** 2022-08-22

**Authors:** Khetsiwe P. Masuku, Anniah Mupawose

**Affiliations:** 1 Department of Speech Pathology, Faculty of Humanities, University of the Witwatersrand, Johannesburg, South Africa

**Keywords:** COVID-19, speech pathology, online clinical training, writing intensive, students

## Abstract

**Background:**

Coronavirus disease 2019 (COVID-19) and the subsequent lockdown altered traditional clinical training for speech language pathology students, thus forcing training institutions to implement innovative and responsive clinical training strategies in the midst of the pandemic. As such, a writing-intense programme was piloted in an online clinical training programme with second-year speech language pathology students.

**Objectives:**

This study explored speech language pathology students’ experiences with a writing programme used during an online clinical training programme implemented during the COVID-19 pandemic.

**Method:**

The study used a qualitative survey design. Purposive convenient sampling was used to recruit 29 second-year speech language pathology students. Online student reflections guided by 10 open-ended questions were used to elicit responses from students. Data were analysed using deductive thematic analysis.

**Results:**

Findings revealed that the written component of the programme facilitated the acquisition of clinical knowledge and improved clinical processes of writing among students. Feedback that students received on their written tasks improved learning. The clinical component of the course enabled students to learn in a less stressful environment and helped them gain confidence in their knowledge and clinical skills. Connectivity challenges and the lack of motivation from some students negatively impacted the programme.

**Conclusion:**

Using a writing programme to clinically train students can have positive effects in applying theory to clinical application because it affords students time to consolidate and process theory with practice as the jump from first year to second year can be cognitively taxing. A writing-intense programme can also improve students’ writing skills.

## Background

Speech language pathology is a degree that comprises a combined theoretical and clinical curriculum. The curriculum is set up to ensure that students can apply theoretical knowledge to a clinical context. For speech language pathology students to effectively apply what they know theoretically to a clinical context, they need to be able to transfer their learning of theory to a clinical context. The ability of students to transfer their learning skills requires developing and using appropriate thinking skills. Writing as a tool promotes critical thinking if the writing assignments go beyond extrapolating the knowledge students have acquired from learning. Extrapolation lends itself to lower-order thinking skills (as per Bloom’s taxonomy), such as remembering, understanding and applying (cited in Armstrong, 2016). When writing is instituted to help students learn the course content on a clinical platform, students are forced to engage in higher-order thinking skills (as per Bloom’s taxonomy), such as analysis, evaluation and synthesis (cited in Armstong, 2016). On a clinical learning platform these skills translate to the student in speech language pathology collating and analysing information, developing an understanding of clients’ needs, planning and implementing intervention and reflecting on client care (Cronin & Graebe, 2018). According to Kamhi (2011) and Coutts and Pillay (2021), these critical thinking skills are the core skills that speech language pathology students need to foster early in the speech language pathology programme to be used in the provision of services and routinely in decision-making. In the context of the coronavirus disease 2019 (COVID-19) pandemic, the clinical training of our speech language pathology students needed to move from the traditional face-to-face method to an online platform. So, the rationale for using a writing-intense programme was to address the clinical outcomes of the clinical course and improve the writing skills of speech language pathology students.

Because of the COVID-19 pandemic, the Department of Speech Language Pathology, like all other departments in the university, had to develop innovative strategies to clinically train our students. We had to train the students in a manner that addressed their gaps in knowledge, addressed their clinical competencies, bridged the gap between theory and practice and improved the writing skills of the students. In our department, second-year students engaged in their first year of clinical training in underserved and under-resourced child language contexts. So, it was imperative that the second-year students obtain a solid foundation for assessing and treating paediatric clients who present with speech, language and literacy disorders. In order to establish a solid foundation as clinical educators we needed to develop an innovative teaching and learning approach that would develop the necessary critical thinking skills needed for clinical decision-making. We were forced to rethink and reimagine the way clinical training would occur (Khoza-Shangase, Moroe, & Neille, 2021), while still maintaining the integrity and continuity of the education process (Ross, 2020) and achieving the required outcomes of the course. We did not want the students disadvantaged in terms of clinical competency and accumulating the necessary number of clinical hours required for students to graduate and register as therapists with the Healthcare Professionals Council of South Africa (HPCSA, 2017).

Previous research has demonstrated that critical thinking skills required for clinical practice can be developed through a number of activities, including simulation in speech language pathology (Ellis, 2017; MacBean, Theodoros, Davidson, & Hill, 2013), questioning across disciplines (Copes, Guenther, Adelung, & Telesca, 2018), peer assessments, for example, in dentistry (Tricio, Woolford, & Escudier, 2016) and using assessments that are culturally and contextually appropriate (Coutts & Pillay, 2021). Literature examining clinical simulations and the development of critical thinking or reasoning skills in speech language pathology revealed that speech language pathology students enjoy simulated cases but need more guidance and feedback on how to interact with cases (Ellis, 2017). MacBean et al. (2013) reported that most Australians find the use of simulated cases important, but the use of simulated cases is limited in speech language pathology programmes. Another study conducted in South Africa by Coutts and Pillay (2021) showed that cultural and contextual factors influence critical thinking skills (clinical decision-making) when conducting bedside dysphagia assessments. In fact, a study by Hayden, Smiley, Alexander, Kardong-Edgren and Jeffries (2014) in nursing showed that there is no significant difference in the development of skills between traditional face-to-face training and clinical simulations. Copes et al. (2018) found that the use of questioning by clinical educators from various disciplines improved students’ critical thinking skills in relation to client management. Although there are studies that have been conducted investigating critical thinking skills and clinical practice, few if any have incorporated the use of a writing-intense approach to develop critical thinking skills for clinical practice on an online platform.

The authors argue that using a writing-intense programme facilitates the critical thinking skills required for learning clinical skills on an online platform. A writing-intense course is defined by Nichols et al. (2019) as:

[*A*]n existing course which is adapted to use writing to deliver course content, where the latter concept is understood as the particular critical thinking skills which the course is designed to teach. (p. 133)

We utilised a writing-intense programme for several reasons. Firstly, the profession of speech language pathology incorporates a great deal of writing. Secondly, writing promotes critical thinking skills, especially if it is linked to the content or context of learning. Thirdly, writing demands that students think ahead, consider their audience and rethink their wording or organisation to ensure that their writing tasks (assessment and intervention reports) meet a specific goal: to inform or explain, evaluate, intepret and/or integrate (Çavdar & Doe, 2012; Nichols et al., 2019). Lastly, writing improves academic literacy and cognitive academic language proficiency (CALP) of students who are first language English and English additional language (EAL) speakers (Dinitz & Harrington, 2014). Academic literacy is the ability to handle the demands of academic language at a tertiary level (Weideman, 2018) and CALP refers to students’ ability to understand and express, in both oral and written forms, concepts and ideas that are relevant to academic success (Cummins, 2008). English additional language students face additional difficulties and stress in academic writing largely because of a lag in their level of language proficiency (Al Fadda, 2012; Dinitz & Harrington, 2014). Therefore, to deal with the difficulties students experience irrespective of language proficiency, in our programme an appropriate critical process of writing was utilised to improve their academic literacy.

The language of learning and teaching in this speech language pathology programme was English. The writing-intense programme utilised in this study was based on the following principles: (1) the use of different types of writing, for example, therapy goals, paragraphs, assessment reports and home programmes; (2) students were given continuous feedback on written assignments from peers and clinical educators; (3) the programme used the jargon or terminology related to the discipline; (4) the written activities contributed significantly to the students’ clinical course mark; (5) the clinical course required a significant amount of writing; and (6) there was meaningful clinical educators–students interaction on each of the written weekly submissions (Hilgers et al., 1995; Nichols et al., 2019). The writing-intense approach is a non-linear activity in which students are required to plan, draft, revise and edit (Hung & Young, 2015). In the process of planning, drafting, revising and editing, students are required to engage in critical thinking skills (Brock, Sanchez, & Sharpe, 2020). Critical thinking skills, such as calculations, interpretations, analysis, synthesis, creating, evaluation, planning and reflection, which are the core skills that speech language pathologists need in the provision of services and decision-making, are required for client care (Brock et al., 2020; Cronin & Graebe, 2018).

The authors set out to answer the following research questions:

1. How did the students experience the written assignments?
2. How did the students experience the clinical course?
3. What did the students experience as challenges of the clinical course?

## Methodology

### Aim

The purpose of this study was to explore the experiences of second-year speech language pathology students on the writing-intense clinical training programme that was implemented during COVID-19 (July–November 2020).

### Methods

Consistent with the study aim, a qualitative survey design was used for the study (Jansen, 2010). A qualitative approach was deemed appropriate because of its flexibility and allowance for a more detailed exploration of the students’ experiences (Berg, 2009). In addition, a qualitative approach produced a broad spectrum of ideas and opinions and different perspectives among the different students (Berg, 2009).

### Participants

A non-probability purposive convenient sampling strategy was utilised to recruit participants (Cresswell & Poth, 2018). A specific inclusion criterion was employed (Leady & Ormrod, 2013). In accordance with the sampling strategy and inclusion criteria, participants had to be registered as second-year speech pathology students, who had participated in the writing-intense online clinical training programme, which was in English. A total of 29 participants who were all women and between the ages of 18 and 20 years (mean age 19 years) participated in the study. The 29 students had done face-to-face clinical practicals at school placements between February and March 2020, and they had also participated in the online clinical training programme between July and November 2020.

### Data collection and analysis

At the end of the 14 weeks of the writing-intense training programme, students were requested to complete an online reflection report on their experiences of the clinical training programme. Student reflections were guided by 10 key open-ended questions (Table 1). Students were given a period of 30 days to complete their reflections. Weekly reminders were sent to students to complete the reflections. Students were informed that their reflections may be used for research purposes, and they were requested to indicate through writing if they consented to their reflections being used for this study. The reflection questions were piloted with two clinical educators who are speech language pathologists. The aim of the pilot study was to determine the content as well as the face validity of the questionnaire, in particular whether the questions asked on the reflection report answered the research question and were ideal as a tool to guide a reflection (Leedy & Ormrod, 2013). The pilot study showed that the questions were appropriate. Transcripts of the students’ responses to the reflection questions were downloaded from the online system and imported into NVivo 1.5.1 qualitative analysis research software and analysed using deductive thematic analysis, following Braun and Clarke’s six steps of thematic analysis (Braun & Clarke, 2006). Themes were informed by the research questions.

**TABLE 1:** Open-ended questions on which students’ reflections were based on.

Number	Question
1.	What did you need to know (prior knowledge and skills) to complete activities for these past two blocks?
2.	What did you learn from activities of these past two blocks?
3.	What clinical activities worked and why?
4.	What clinical activities did not work and why?
5.	How did you feel about what you achieved or experienced in the past two blocks?
6.	What have you learnt about your clinical skills and/or about yourself through the activities of the past two blocks?
7.	Which of your clinical skills do you think you need to further develop, and what will you do to develop these skills further?
8.	What about the written activities worked and why?
9.	What about the written activities did not work and why?
10.	If this structure used for blocks 3 and 4 were to be used as part of clinical training, how would you suggest that it is better improved?

### Trustworthiness

To ensure trustworthiness, Shenton’s (2004) strategies were used. In accordance with the strategies, the 10 reflection questions were piloted to determine content and face validity. Even though all students were invited to submit reflections, participation in the study was still voluntary and was not graded; thus, only responses from students who wanted to be part of the study are included in the analysis. Collaborative coding was employed to mitigate any research bias. The authors have provided descriptions of the parameters of the study and have also stipulated its limitations.

### Ethical considerations

Ethical clearance to conduct the study was obtained from relevant authorities (clearance number: H20/05/45). Permission was requested from students to use their reflections as part of the study. All 29 students gave written permission and consent for their reflections to be used for this study.

## Findings

Eight themes that describe how second-year speech pathology students in their first year of clinical practice experienced the writing-intensive clinical online training programme implemented during the COVID-19 pandemic emerged from the data. The themes and quotes from students are presented in Table 2 and further discussed in the discussion section. Students reported that the written assignments improved their clinical knowledge. The feedback that students received from their peers and clinical educators as well as the discussions that they engaged in based on their written assignments improved students’ learning. Written assignments further improved the students’ critical processes by teaching students the mechanics of academic writing. Students reported that the clinical component of the writing-intense online training programme enabled them to learn clinical knowledge and skills and improved their confidence in a less stressful environment because it was simulated. The online nature of the programme presented with challenges such as connectivity (data and infrastructure) and inconsistent participation from students, which negatively affected the programme.

**TABLE 2:** Themes from students’ reflections.

Research question	Themes	Excerpts from participants
How did the students experience the written assignments?	Improved clinical knowledge	‘I can confidently write SMART goals for each type of language domain, because I understand what each [language] domain is… and why it is relevant.’ (Student # 6; Female; 20 years old)
		‘Grouping the different subtests into semantics, syntax, etc., tabulating the client’s strengths and weaknesses and writing paragraphs worked because breaking it down helped with understanding each component and making meaning of it.’ (Student # 1; Female; 21 years old)
		‘I think the paragraphs and analysing the results per section, such as focusing on semantics one week, then syntax the next week, really helped. It allowed me to further research and learn about each aspect of language and literacy. It helped me learn more about [aspects of language] when they were focused on individually.’ (Student # 12; Female; 21 years old)
	Feedback and discussions	‘I enjoyed the focus on the goals where we would receive feedback to learn how to write professional language, literacy and auditory processing goals.’ (Student #1; Female; 21 years old)
		‘Having the supervisors meet with us and discuss cases was a huge game changer. I learnt the most from them and from the interaction from my peers. And I think because I enjoyed the engaging aspect of it, my motivation and excitement levels for blocks 3 and 4 really improved.’ (Student #13; Female; 20 years old)
	Critical processes of writing	‘But in general, I think that the paragraphs were good in that they forced me to structure my academic writing in a better manner and analyse language samples or assessment results critically and in more detail.’ (Student #6; Female; 20 years old)
		‘Doing draft work really helped as you could learn more effectively, get feedback and change mistakes much quicker than the times where we only had one final submission.’ (Student #8; Female; 20 years old)
How did the students experience the clinical course?	Clinical learning environment	‘I found that, at the beginning of the year, learning skills at the same time we were supposed to apply them with the client was hugely stressful, this way [online] was less stressful.’ (Student #2; Female; 20 years old)
		‘I felt that this way of learning [online] was much less stressful and gave us time to make mistakes without having negative ramifications on an actual client’. … in the beginning of the year I felt negative about going to pracs because I did not know what I was doing, and had the pressure of having a client…[Now] I feel a lot better in terms of being equipped. This way [online] of learning has been a lot less stressful in that we do not have clients.’ (Student #4; Female; 20 years old)
	Confidence	‘I feel much more confident about being able to assess and provide intervention to (to a lesser extent) clients.’ (Student # 14; Female; 21 years old)
		‘I think the past blocks have made me more comfortable in identifying the areas of communication children have difficulty…. either formal or informal assessment.’ (Student #15; Female; 20 years old)
		‘I’ve realised I need to have more confidence in myself. I’ve noticed that I can have more confidence in my clinical skill [s] even if it is not perfect, which also took me a while to know that [imperfection] is normal.’ (Student #13; Female; 20 years old)
	Knowledge and skills	My skills are getting better and I’m learning new ways of doing things and building on the skills where I can. ‘I think my clinical skills have improved, as my knowledge on each concept has improved and developed and I now have many new ideas for activities, new techniques that are my own ideas as well.’ (Student #11; Female; 20 years old)
		‘The guidance and constructive feedback from my clinical supervisor has allowed me to gain new knowledge and skills that I hope to use throughout my future working.’ (Student #9; Female; 20 years old)
What are the challenges experienced by students with the training programme?	Connectivity challenges	‘Sometimes video calls would not work due to an array of factors, such as connection issues.’ (Student #5; Female; 20 years old)
		‘My area sometimes does not have electricity for days, so I would struggle to participate.’ (Student #12; Female; 21 years old)
		‘The university gave us data, but it is really not enough for everything, also there is night and day data, we really don’t have discussions with supervisors at night now.’ (Student #10; Female; 20 years old)
	Participation	‘But also things like not everyone answering in equal amounts during the call affected the discussion.’ (Student #2; Female; 20 years old)
		‘Sometimes you would evaluate your peer’s work, but then they would not evaluate yours or just do the bare minimum.’ (Student #5; Female; 20 years old)

## Discussion

One of the outcomes of the speech language pathology degree is to develop the ability of students to acquire theoretical knowledge about communication disorders as well as to apply that knowledge clinically in the management of patients presenting with communication disorders. For students to be able to do this, they require critical thinking skills. Critical thinking skills are fundamental to decision-making in the clinical service provision in speech language pathology (Kamhi, 2011). One of the ways in which critical thinking skills can be promoted is through engaging students in writing-intense tasks that require them to use higher-order cognitive skills, such as collecting information; developing an understanding of clients’ needs; and then planning, directing, performing and reflecting on appropriate client care (Cronin & Graebe, 2018). During the lockdown instituted because of the COVID-19 pandemic, face-to-face clinical teaching was suspended. Therefore, a writing-intense online clinical programme was implemented with second-year speech language pathology students between July and November 2020. The study explored, through student reflections, how the 29 second-year speech language pathology students who were registered and participated in the course experienced the written assignments and the clinical component as well as what they experienced as challenges. Findings (themes) from students’ reflections are presented in Table 2 and are discussed below according to the different research questions.

## How did the students perceive the written assignments?

Findings from students’ reflections revealed that engaging in writing-intense tasks facilitated the students’ understanding of theoretical and clinical content. This finding supports the ‘writing to learn’ principle of writing-intense courses documented in McLeod and Miraglia (2011) and Thaiss et al. (2012), where the authors in the mentioned studies argue that the writing to learn principle of writing-intense courses assists students to learn discipline-specific course content through writing, therefore improving their learning and marks. Although the study at hand is not reporting on grades, the positive effects of the writing activities on students’ learning came across strongly in the student responses. International studies on writing-intense programmes further present that using a writing-intense approach is an ideal method to promote students’ learning about difficult and unfamiliar course-related concepts (Belting & Lanninh, 2017; Peters et al., 2019).

Students in the study also reported that the writing tasks promoted opportunities for engagements between students and clinical educators through peer and clinical educator feedback as well as through online discussions, which the students appreciated. Feedback and discussions forced students to move from simplistic ways of understanding and interpreting content and clinical cases to a more nuanced and complex understanding, thus aligning with more deep as opposed to surface learning. Findings from the study support the conclusion stated by Brock et al. (2020) that feedback and discussion sessions derived from multiple drafts of writing on the same topic form an integral part of a writing-intense course. Feedback and discussion in a writing-intense course not only facilitate the social aspect of learning, but also challenge students to opportunities to consider, develop and rethink initial responses, therefore facilitating integration and the development of critical thinking skills (Bean, 2011; Brock et al., 2020; Cronin & Graebe, 2018).

Students further perceived the writing assignments to have encouraged the critical processes of writing. Students reported that the writing component of the clinical programme was significant in teaching them the structure of drafting a written academic piece and their ability to provide a critical and detailed reporting of clinical findings. The provision of a structure (e.g. a thesis statement, supporting details and conclusion), a marking rubric for written paragraphs and allowing multiple drafts of written work was a good way of scaffolding students’ written work. The findings of this study corroborate those of Marshall and Marr (2018) and Peters et al. (2019), where the authors present that one of the benefits of writing-intense activities was to improve the writing skills of students, skills that are necessary for future academic careers as well as employment. Written work is a crucial component in the speech language pathology profession, as speech pathology students and clinicians are required to write clinical assessment and intervention reports, research reports and clinical home programmes.

## How did the students perceive the clinical course?

Students in this study reported that the clinical component of the online training programme promoted the learning of clinical work in a less stressful environment. Students found that having to start clinical work directly with real-life patients especially in their first year of clinical work caused them stress and anxiety. Therefore, using the template of a simulated approach where the written activities are the focus is an option that could be used to ease students into clinical work, especially in their first year of clinical practicals. This theme corroborates that of Mohammadi, Tourdeh and Ebrahimian (2019) in a study where they describe the effect of simulation-based training methods on the psychological health promotion of students during their educational internship. Findings from Mohammadi et al.’s (2019) study revealed that simulation is one of the strategies that is suggested and used to reduce stress and anxiety in clinical student education. Tension and anxiety in clinical training should ideally be reduced as far as possible because they affect students’ proper clinical training efficiency, academic performance, critical thinking, learning outcomes and cognitive appraisal (Saravanan & Wilks, 2014).

The clinical component of the training programme was reported to have facilitated confidence in the students’ knowledge and skills on assessing and managing clients with language and literacy difficulties. Singh et al. (2020) in a study that looked at significant application of virtual reality cases during COVID-19, albeit in the medical fraternity, highlighted an increase in self-confidence, positive psychological affects, improved teamwork, increased skills of students and overall performance, which are factors also mentioned by students in this study. Improved knowledge and skills in assessing and managing language and literacy in paediatric populations were attributed to constructive feedback received from peers as well as from clinical educators. Copes et al. (2018) argued that probing by clinical educators during student supervision enhanced students’ critical thinking skills with respect to client management. In another study by Williams, Dudding and Ondo (2013), cited in Ellis (2017), the authors evaluated the role of feedback in virtual human experiences in their graduate speech-language pathology courses. In Williams et al.’s (2013) study cited in Ellis (2017), students who had received constant feedback performed much better than those who did not. Similarly, Ellis et al. (2017) reported that speech language pathology students enjoy simulated cases but need more guidance and feedback on how to interact with cases. We suggest that using a writing-intense approach provides an alternative method of providing guidance and feedback to students.

## What were the challenges experienced by students with the training programme?

Even though the students had mostly positive feedback on the writing-intense clinical training programme, some students battled with keeping on track with tasks because of the online nature of the clinical training. Some students in the course lived in remote areas with poor connectivity and limited access to data, which are factors that negatively influenced their participation. Mishra, Gupta and Shree (2020) and Pillay and Agherdien (2021) conducted studies in low- and middle-income countries, India and South Africa, respectively, and both acknowledged that in these contexts the digital divide is evident. While some students have the necessary resources to successfully engage in online teaching and learning, other students experience challenges because of interrupted electricity connection, intermittent signal issues and unaffordable data costs. Commitment to participation in group activities was not the same for all students, therefore affecting the learning process. Mishra et al. (2020) reported similar challenges, which they attributed to a certain degree a lack of motivation and commitment.

## Conclusion

This article only focuses on reflections of students to evaluate the programme. The voices of clinical educators and course coordinators are missing. However, as this study is a pilot study, starting with the experiences of students was deemed a good starting point. Future research needs to include the perspectives of clinical educators in using a writing-intense programme on an online platform. There is an assumption that the academic literacy of the students improved on this platform. We did not particularly delve into the CALP of the students, which is an issue that can be investigated in future research. Lastly, the students may not have responded to all the questions and therefore inter-student or intra-student comparisons are not feasible (Eadie et al., 2006).

The strength of this study is that it provides an alternative method to the clinical training of speech language pathology students. The study strongly suggests that online writing-intense programmes can facilitate critical thinking skills that are important in decision-making in speech language pathology clinical practice. The structure of the writing-intense programme, for example, providing a thesis statement, supporting detail and conclusion, provides students with support and scaffolding for written tasks. This structure can be generalised to any clinical written work, for example, client reports and home programmes. The writing assignments, feedback from peers and clinical educators and the revision processes fostered more deep structures of writing (Simon, 2013), as writing was not used to access knowledge but to rather encourage critical thinking. The writing assignments, feedback from peers and clinical educators and the revision processes also facilitated the learning of clinical knowledge and applying theory to clinical practice. Incorporating an online clinical writing programme, which follows the template of simulated learning, gave an opportunity for students to consolidate and process theory with practice, as the move from first year to second year can be cognitively challenging for students. An online clinical writing programme presented before students see real-life patients decreases the anxiety and stress of treating real-life patients for the first time. When implementing an online programme, such as this one, consideration for access to resources, such as data, devices and electricity, should be done, so that all students can actively participate in the programme. Lastly, in formulating the written assignments and activities, consideration needs to be done in a manner that does not overburden students but rather facilitates critical thinking.

## References

[CIT0001] Al Fadda, H. (2012). Difficulties in academic writing. From the perspectives of King Saud University post graduate students. *English Language Teaching*, 5(3), 123–130. 10.5539/elt.v5n3p123

[CIT0002] Armstrong, P. (2016). *Bloom’s taxonomy*. Vanderbilt University. Retrieved from https://cft.vanderbilt.edu/guides-sub-pages/blooms-taxonomy/

[CIT0003] Bean, J.C. (2011). *Engaging ideas: The professor’s guide to integrating writing, critical thinking, and active learning in the classroom*. San Francisco, CA: Jossey-Bass.

[CIT0004] Belting, B., & Lannin, A. (2017). University of Missouri campus writing program. In B.S. Finer & J. White-Farnham (Eds.), *Writing program architecture: Thirty cases for reference and research* (pp. 155–169). Boulder, CO: University Press of Colorado.

[CIT0005] Berg, B. (2009). *Qualitative research methods for the social sciences*. Boston, MA: Pearsons Education, Inc.

[CIT0006] Braun, V., & Clarke, V. (2006). Using thematic analysis in psychology. *Qualitative Research in Psychology*, 3(2), 77–101. 10.1191/1478088706qp063oa

[CIT0007] Brock, C., Sanchez, N., & Sharpe, D.L. (2020). Pen as a bridge: Instructor perspectives on incorporating diversity and inclusion in writing intensive courses. *Teaching in Higher Education*, 25(8), 992–1009. 10.1080/13562517.2019.1628733

[CIT0008] Çavdar, G., & Doe, S. (2012). Learning through writing: Teaching critical thinking skills in writing assignments. *PS: Political Science & Politics*, 45(2), 298–306. 10.1017/S1049096511002137

[CIT0009] Copes, A., Guenther, L., Adelung, M.R., & Telesca, L. (2018). In-TICE an integrated tool for professional educators. *Journal of Allied Health*, 47(3), e83. 30194835

[CIT0010] Coutts, K., & Pillay, M. (2021). Decision making and the bedside assessment: The speech language therapists’ thinking when making a diagnosis at the bed. *South African Journal of Communication Disorders*, 68(1), 1–8. 10.4102/sajcd.v68i1.790 PMC8252154 34212747

[CIT0011] Cresswell, J., & Poth, C. (2018). *Qualitative inquiry and research design*. Los Angeles and Washington DC: Sage.

[CIT0012] Cronin, A., & Graebe, G. (2018). *Clinical reasoning in occupational therapy*. Bethesda, MA: AOTA.

[CIT0013] Cummins, J. (2008). BICS and CALP: Empirical and theoretical status of the distinction. *Encyclopedia of Language and Education*, 2(2), 71–83. 10.1007/978-0-387-30424-3_36

[CIT0014] Dinitz, S., & Harrington, S. (2014). The role of disciplinary expertise in shaping writing tutorials. *The Writing Center Journal*, 33(2), 73–98.

[CIT0015] Eadie, T.L., Yorkston, K.M., Dudgeon, B.J., Deitz, J.C., Baylor, C.R., Miller, R.M., … Amtmann, D. (2006). Measuring communicative participation: A review of self-report instruments in speech language pathology. *American Journal Speech Language Pathology*, 15(4), 307–320. 10.1044/1058-0360(2006/030) PMC2649949 17102143

[CIT0016] Ellis, C.M. (2017). Using simulation and critical thinking in speech-language pathology: A university case study. *Journal of Human Services: Training, Research, and Practice*, 2(2), 6.

[CIT0017] Hayden, J., Smiley, R., Alexander, M., Kardong-Edgren, S., & Jeffries, P. (2014). The NCSBN national simulation study: A longitudinal, randomized, controlled study replacing clinical hours with simulation in prelicensure nursing education. *The Journal of Nursing Regulation*, 5(2), 1–64. 10.1016/S2155-8256(15)30062-4

[CIT0018] Health Professions Council of South Africa (HPCSA). (2017). Regulations defining the scope of the profession of speech language therapy. Department of Health. Retrieved from https://www.hpcsa.co.za/Uploads/SLH/Rules%20and%20Regulations/Regulations_defining_the_scope_of_the_profession_of_Speech_Language_Therapy%20.pdf

[CIT0019] Hilgers, T.L., Bayer, A.S., Stitt-Bergh, M., & Taniguchi, M. (1995). Doing more than ‘thinning out the herd’: How eighty-two college seniors perceived writing-intensive classes. *Research in the Teaching of English*, 29, 59–87.

[CIT0020] Hung, H.C., & Young, S.S.C. (2015). The effectiveness of adopting E-readers to facilitate EFL students’ process-based academic writing. *Journal of Educational Technology & Society*, 18(1), 250–263.

[CIT0021] Jansen, H. (2010). The logic of qualitative survey research and its position in the field of social research methods. *Forum Qualitative Sozialforschung/Forum: Qualitative Social Research*, 11(2). 10.17169/FQS-11.2.1450

[CIT0022] Kamhi, A.G. (2011). Balancing certainty and uncertainty in clinical practice. *Language, Speech, and Hearing Services in Schools*, 42(1), 59–64. 19833828 10.1044/0161-1461(2009/09-0034)

[CIT0023] Khoza-Shangase, K., Moroe, N., & Neille, J. (2021). Speech-language pathology and audiology in South Africa: Clinical training and service in the era of Covid-19. *International Journal of Telerehabilitation*, 13(1), e6376. 10.5195/ijt.2021.6376 34345349 PMC8287713

[CIT0024] Leedy, P.D., & Ormrod, J.E. (2013). *Practical research: Planning and design* (10th ed.). Boston, MA: Pearson Education.

[CIT0025] Leedy, P.D., & Ormrod, J.E. (2013). The nature and tools of research. In *Practical research: Planning and design* (vol. 1, pp. 1–26). New Jersey: Pearson.

[CIT0026] MacBean, N., Theodoros, D., Davidson, B., & Hill, A.E. (2013). Simulated learning environments in speech-language pathology: An Australian response. *International Journal of Speech-Language Pathology*, 15(3), 345–357. 10.3109/17549507.2013.779024 23586581

[CIT0027] Marshall, S., & Marr, J.W. (2018). Teaching multilingual learners in Canadian writing-intensive classrooms: Pedagogy, binaries, and conflicting identities. *Journal of Second Language Writing*, 40, 32–43. 10.1016/j.jslw.2018.01.002

[CIT0028] McLeod, S.H., & Miraglia, E. (2011). Writing across the curriculum in a time of change. In S.H. McLeod, K. Miralgia, M. Soven, & C. Thaiss (Eds.), *WAC for the new millennium: Strategies for continuing writing-across-the-curriculum programs* (pp. 1–27). Urbana, IL: National Council of Teachers of English.

[CIT0029] Mishra, L., Gupta, T., & Shree, A. (2020). Online teaching-learning in higher education during lockdown period of Covid-19 pandemic. *International Journal of Educational Research Open*, 1, 100012. 10.1016/j.ijedro.2020.100012 35059663 PMC7832355

[CIT0030] Mohammadi, G., Tourdeh, M., & Ebrahimian, A. (2019). Effect of simulation-based training method on the psychological health promotion in operating room students during the educational internship. *Journal of Education and Health Promotion*, 8, 172. 31867357 10.4103/jehp.jehp_106_19 PMC6796314

[CIT0031] Nichols, P., Erasmus, E., Ntsepo, N., Mlahleki, L., Mabalane, K., Ngobeni, K., … Ckool, L.I. (2019). Writing within simultaneity: A reflexive progress report through letter from Wits writing programme. *Stellenbosch Papers in Linguistics Plus*, 57, 131–147. 10.5842/57-0-814

[CIT0032] Peters, A.W., Tisdale, V.A., & Swinton, D.J. (2019). *High impact practice: Writing intensive courses*. Retrieved from https://cowyamp.colostate.edu/high-impact-practice-writing-intensive-courses/

[CIT0033] Pillay, R., & Agherdien, N. (2021). Inclusivity, equality and equity: Student (em) power (ment) through ICT mediated learning. In M. Maguvhe, R.S. Mphahlele, & S. Moonsamy (Eds.), *Empowering students and maximising inclusiveness and equality through ICT* (Vol. 49, pp. 11–26). Lieden and Boston: Brill Sense.

[CIT0034] Ross, D.A. (2020). Creating a ‘quarantine curriculum’ to enhance teaching and learning during the COVID-19 pandemic. *Academic Medicine*, 95(8), 1125–1126. 10.1097/ACM.0000000000003424 PMC7179056 32744816

[CIT0035] Saravanan, C., & Wilks, R. (2014). Medical students’ experience of and reaction to stress: The role of depression and anxiety. *Scientific World Journal*, 2014, 737382. 10.1155/2014/737382 24688425 PMC3929074

[CIT0036] Shenton, A.K. (2004). Strategies for ensuring trustworthiness in qualitative research projects. *Education for Information*, 22(2), 63–75. 10.3233/EFI-2004-22201

[CIT0037] Simon, R. (2013). ‘Starting with what is’: Exploring response and responsibility to student writing through collaborative inquiry. *English Education*, 45(2), 115–146.

[CIT0038] Singh, R.P., Javaid, M., Kataria, R., Tyagi, M., Haleem, A., & Suman, R. (2020). Significant applications of virtual reality for Covid-19 pandemic. *Diabetes & Metabolic Syndrome: Clinical Research & Reviews*, 14(4), 661–664. 10.1016/j.dsx.2020.05.011 PMC7214336 32438329

[CIT0039] Thaiss, C. (2012). Origins, aims, and uses of writing programs worldwide: Profiles of academic writing in many places. In *Writing programs worldwide: Profiles of academic writing in many places* (pp. 5–22). Perspectives on Writing. Fort Collins.

[CIT0040] Tricio, J.A., Woolford, M.J., & Escudier, M.P. (2016). Fostering dental students’ academic achievements and reflection skills through clinical peer assessment and feedback. *Journal of Dental Education*, 80(8), 914–923. 10.1002/j.0022-0337.2016.80.8.tb06171.x 27480702

[CIT0041] Weideman, A. (2018). *Academic literacy: Five new tests*. Bloemfontein: Geronimo Distribution.

[CIT0042] Williams, S., Dudding, C., & Ondo, K. (2013). *Simulations & beyond: Practical considerations for getting started*. [Convention handout]. Retrieved from http://find.asha.org:8085/asha#q=Stacy%20Williams&sort=relevancy&f:@sysfiletype=[pdf]

